# Non specific pattern of lung function in a respiratory physiology unit: causes and prevalence: results of an observational cross-sectional and longitudinal study

**DOI:** 10.1186/1471-2466-14-148

**Published:** 2014-09-19

**Authors:** Brigitte Chevalier-Bidaud, Karine Gillet-Juvin, Etienne Callens, Romain Chenu, Sémia Graba, Mohamed Essalhi, Christophe Delclaux

**Affiliations:** AP-HP, Hôpital européen Georges-Pompidou, Unité d’Épidémiologie et de Recherche Clinique, 75015 Paris, France; AP-HP, Hôpital européen Georges-Pompidou, Service de Physiologie – Clinique de la Dyspnée, 75015 Paris, France; AP-HP, Hôpital européen Georges-Pompidou, Service de Pneumologie, 75015 Paris, France; Sorbonne Paris Cité, Faculté de médecine, Université Paris Descartes, 75006 Paris, France; Sorbonne Paris Cité, Université Paris Descartes, Paris, 75014 EA2511 France; CIC 9201 Plurithématique, Hôpital Européen Georges Pompidou, 75015 Paris, France

**Keywords:** Asthma, Bronchiectasis, COPD, Emphysema, Pulmonary hypertension, Sarcoidosis, Small airway obstructive syndrome, Non specific pattern of lung function

## Abstract

**Background:**

ATS/ERS Task Force has highlighted that special attention must be paid when FEV_1_ and FVC are concomitantly decreased (<5^th^ percentile) and the FEV_1_/FVC ratio is normal (>5^th^ percentile) because a possible cause of this non specific pattern (NSP) is collapse of small airways with normal TLC measured by body plethysmography (>5^th^ percentile). Our objectives were to determine the main lung diseases associated with this pattern recorded prospectively in a lung function testing (LFT) unit, the prevalence of this pattern in our LFT and among the diseases identified, and its development.

**Methods:**

Observational study of routinely collected data selected from our Clinical Database Warehouse.

**Results:**

The prevalence of NSP was 841/12 775 tests (6.6%, 95% CI: 6.2 to 7.0%). NSP was mainly associated with seven lung diseases: asthma (prevalence of NSP among asthmatics: 12.6%), COPD/emphysema (prevalence 8.6%), bronchiectasis (12.8%), sarcoidosis (10.7%), interstitial pneumonia (4.0%), pulmonary hypertension (8.9%) and bilateral lung transplantation for cystic fibrosis (36.0%). LFT measurements were described in 185 patients with NSP and indisputable nonoverlapping causes. A moderate defect (FEV_1_: 66 ± 9% predicted) with mild lung hyperinflation (FRC: 111 ± 27%, RV: 131 ± 33% predicted: suggesting distal airway obstruction) was evidenced whatever the underlying cause. A long term stability of NSP was evidenced in 130/185 patients (70% 95% CI: 64 to 77%).

**Conclusions:**

NSP is observed in asthma, COPD/emphysema, bronchiectasis, sarcoidosis, pulmonary hypertension, interstitial pneumonia and after bilateral lung transplantation and remains stable in the majority of patients.

## Background

An obstructive ventilatory defect is a disproportionate reduction in maximal airflow from the lung in relation to the maximal volume (*i.e.* vital capacity, VC) that can be displaced from the lung [1]. It is defined by a reduced Forced Expiratory Volume in 1 second (FEV_1_)/VC ratio below the 5^th^ percentile of the predicted value. As stated by the ATS/ERS task force [1], “special attention must be paid when FEV_1_ and forced VC (FVC) are concomitantly decreased and the FEV_1_/FVC ratio is normal or almost normal […]. A possible cause of this pattern is patchy collapse of small airways early in exhalation. Under these conditions, total lung capacity (TLC) may be normal, but residual volume (RV) is ordinarily increased. ” This pattern has been named as small airways obstruction syndrome by Stanescu [2], and has initially been described in small series of adults with emphysema, small airways diseases, bronchial asthma [2] and also in asthmatic children [3]. Hyatt and colleagues identified more recently the medical conditions associated with this pattern from a database of lung function tests (LFT) results and medical records [4]. These authors showed that 68% of the patients were suffering from airway disease (including asthma, chronic obstructive pulmonary disease [COPD] and bronchiectasis), while the others suffered from restricted expansion of the thorax or the lung [4]. Consequently, they preferred to define this pattern as a non specific pattern (NSP).

The objective of our observational study using routinely collected data were to confirm the results of Hyatt and colleagues (main lung diseases associated with this syndrome), to further assess the prevalence of this syndrome in these diseases and to describe the follow-up of this NSP as done by the same research group [5]. These objectives have practical consequences for lung function testing units because if the prevalence of the syndrome is “significant” it is a plea for systematic absolute lung volume measurement when a reduction of FEV_1_ with a normal FEV_1_/FVC ratio is evidenced.

## Methods

Reporting is in accordance with STROBE Statement (http://www.strobe-statement.org/). Unfortunately, the statements of the REporting of studies Conducted using Observational Routinely collected Data (RECORD, http://www.strobe-statement.org/) are not available.

### Design

**Figure 1:**
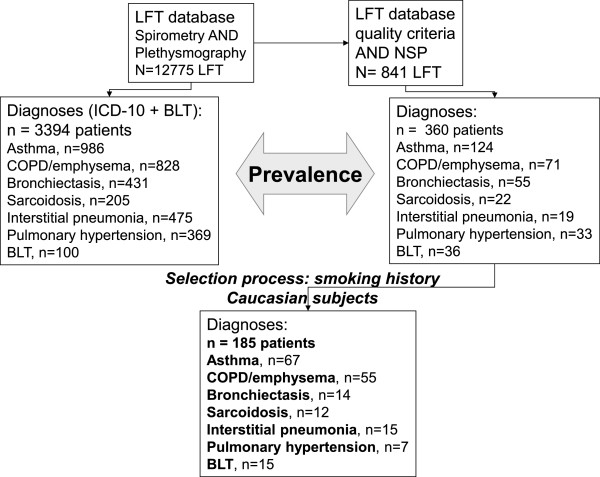
**Selection of the patients and prevalence estimation.** LFT: lung function tests; NSP: Non Specific Pattern; COPD: chronic obstructive pulmonary disease; BLT: bilateral lung transplantation (that was identified in the Clinical Database Warehouse by a specific CCAM code). PY: Pack-years for smoking history. The process of data selection is described in the Methods section. On the right side of the flow-chart diagram, the selection process of the 185 patients with NSP described in Table 2 is showed. The first step allowed the selection of patients instead of LFT (from 841 LFT to 360 patients): 30 patients with miscellaneous diseases (see Methods) were excluded, and 360 patients were selected (451 tests were follow-up LFT). All these 360 patients had the “NSP” diagnosis selected by the physician in charge of the medical report, based on the results of LFT showing the predicted value of each parameter and their 5^th^ percentile (95^th^ percentile for absolute lung volumes, additionally) based on normative equations for spirometry and lung volumes with correction for ethnicity [13, 14]. Consequently, the prevalence of NSP in each lung disease was calculated from this sample. The next step was conducted to describe lung function test results from a highly selected subgroup with indisputable diagnoses. For the purpose of the study a more recent Caucasian set of predicted values for spirometry was used [12] allowing the calculation of Z-scores. After the exclusion of non Caucasian subjects and patients with a smoking history ≥ 15 pack-year for non COPD/emphysema diagnoses, 185 / 360 patients were available for the final description.

Since January 2007, explicit procedures for the interpretation of LFT using a standardised report based on interpretative strategies of the ATS/ERS task force, including NSP definition (FEV_1_ and FVC < 5^th^ percentile with FEV_1_/FVC > 5^th^ percentile and TLC measured by body plethysmography > 5^th^ percentile [1]), have been implemented in our unit. The physicians who are responsible for ordering tests have to state the clinical question to be answered and the physicians who are responsible for interpreting the results of LFT further ask patients why they were sent for testing, record ethnicity (mandatory), respiratory symptoms (including Medical Research Council [MRC] dyspnea score 1 to 5, mandatory), as well as smoking status (mandatory), the type and dosage of any medication that may alter lung function (tests are performed while the patient takes his/her usual treatment excepting methacholine challenge test), including when the drugs were last administered, and quality criteria of LFT in our database allowing a standardized LFT report (mandatory: see Figure 1).

Our first objective was to identify the main lung diseases associated with the recording of NSP. It has to be stated that a non reduced TLC measured by body plethysmography was an inclusion criterion according to guidelines [1]; thus patients with restricted expansion of the lung and the chest wall were not retrieved even if our clinical experience shows that some patients with interstitial pneumonia may exhibit a more significant reduction of FEV_1_ and FVC (as compared with TLC reduction) with normal FEV_1_/FVC (complex restrictive pattern [6]).

Due to the observational design of the study using routinely collected data only indisputable diagnoses were selected by the agreement between the diagnosis reported (1) by the LFT physician making the report (LFT Database), and (2) by the code in the Clinical Database Warehouse (CDW, including International Classification of Diseases [ICD-10, recorded by the staff of pulmonologists] and CCAM French public health insurance codes). Furthermore, the diagnoses were controlled for tobacco exposure. We thus selected patients (from January, 2007 to December, 2012) based on their smoking history: COPD were smokers ≥ 15 pack-year (repeated LFT allowed to ensure conventional criteria of COPD, namely post-bronchodilator FEV_1_/FVC < 70%; emphysema, especially centrilobular, was deemed to be present in presence of associated hyperinflation [any increase in static lung volumes]), while other diagnoses were associated with a smoking history < 15 pack-year. If the patient had undergone multiple LFT over this 6-year period, we used only the measurements from their first visit for the cross-sectional assessment. Obviously, for COPD patients, NSP diagnosis is incompatible with COPD diagnosis since FEV_1_/FVC is > 70%. Thus, only COPD patients with transient NSP were retrieved [5]. Finally patients with multiple lung disease diagnoses were excluded.

The second objective was to evaluate the prevalence of the NSP in our LFT unit and among the main lung diseases that have been identified, using the number of NSP over all tests recorded in the LFT database and the number of NSP in each lung disease over the number of patients with each disease having had LFT, respectively.

The third objective was to assess the long term follow-up of the patients exhibiting NSP.

The selection process is summarized in Figure 1, it satisfied to the following steps:

1. For the main objective (main lung diseases associated with NSP)

- extraction of all LFT recorded as NSP in the LFT database
- selection of the first LFT for each patient with NSP- identification of main lung diseases associated with NSP (LFT database): recorded diagnosis for at least 5 patients (miscellaneous diseases were excluded, see Figure 1 legend)
- confirmation of the diagnosis by pulmonologists (ICD-10 database)
- selection of Caucasian subjects with NSP
- extraction of LFT raw data of patients with NSP
- calculation of their percentages of predicted values and Z-scores (the normative equations used are for Caucasian subjects)

2. For the secondary objective (prevalence of NSP)

- number of patients with NSP over the number of patients with LFT including both spirometry and plethysmography (prevalence of NSP in our LFT, whatever the associated disease)
- number of patients with NSP and one specific lung disease over the number of patients with LFT (spirometry plus plethysmography) and this specific lung disease recorded in ICD-10 database (prevalence of NSP among each lung disease).

3. For the follow-up study

- patients with an NSP and at least one follow-up LFT 6 months or more after the initial assessment were identified and included in the follow-up study.

Lung function testing was done by four experienced pulmonary technologists. The tests included spirometry, body plethysmography with airway resistance (Raw using sRaw total [7]) and sometimes lung diffusion for carbon monoxide (DLCO) measurements using an automated system (MasterScreen, Jaeger, CareFusion®, San Diego, California: two apparatus, unchanged over the six-years of the survey) according to the ATS/ERS task force performed during the same visit [1, 8–11]. After raw data extraction, predicted values of Caucasians were calculated for spirometry using Stanojevic and colleagues reference values [12], while values for slow VC, FEV_1_/slow VC and body plethysmography were those routinely used in our testing unit [13, 14].

This was an observational study in which informed consent was obtained from all subjects, and the database was declared to the French regulatory agency for computer data (Commission Nationale de l’Informatique et des Libertés, n° 1391593 v0). Verbal consent was obtained according to the French Law, which was confirmed by The Institutional Review Board of the French learned society for respiratory medicine (SPLF: CEPRO 2013–010).

### Statistics

Results are expressed as mean ± SD or percentages with 95% confidence interval (CI) with the exception of MRC score. Comparison of TLC obtained by plethymography and alveolar volume (VA) obtained during DLCO measurement used the paired *t* test. Comparisons used Kruskall Wallis test for continuous variables and chi-2 test for qualitative variables (see Table 1). A p value < 0.05 was deemed significant.

**Table 1:** **Characteristics of the 185 patients with Non Specific Pattern of Lung Function**

Characteristic	185 patients with NSP
Mean ± SD (except when stated) or n	
Sex, Female/Male	99/86
Age, years	57 ± 19
BMI, kg.m^−2^	26.9 ± 8.4
BMI ≥ 30/25 to 30/< 25 kg.m^−2^	49/55/81
Smoking history	
Never smokers, n	105
Ex-smokers, n	48
Current smokers, n	32
Pack-year, median [interquartile]	0 [0 ; 35]
MRC score, median [interquartile]	2.0 [1.0 ; 2.0]
FEV_1_, L	1.96 ± 0.68
FEV_1_,% predicted [12]	66 ± 9
FEV_1_, z-score	- 2.51 ± 0.61
FVC, L	2.66 ± 0.82
FVC,% predicted [12]	67 ± 9
FVC, z-score	- 2.58 ± 0.71
FEV_1_/FVC	0.77 ± 0.08
FEV_1_/FVC,% predicted [12]	99 ± 9
FEV_1_/FVC, z-score	- 0.24 ± 0.98
Slow inspiratory VC, L	2.71 ± 0.79
Slow inspiratory VC,% predicted [13]	76 ± 11
FEV_1_ / Slow inspiratory VC	0.75 ± 0.08
FEV_1_ / Slow inspiratory VC, 5^th^ percentile [13]	0.68 ± 0.04
TLC, L	5.49 ± 1.19
TLC,% predicted [14]	96 ± 12
TLC, 5^th^ percentile	4.66 ± 1.07
FRC, L	3.46 ± 0.92
FRC,% predicted [14]	111 ± 27
RV, L	2.68 ± 0.85
RV,% predicted [14]	131 ± 33
Raw_tot_, kPa.s.L^−1^	0.46 ± 0.28
Raw_tot_,% predicted	154 ± 99
Specific Raw_tot_, kPa.s	1.60 ± 0.98
Specific Raw_tot_,% predicted	166 ± 101
VA, L (n patients with measurement)*	4.01 ± 0.90 (62)
VA/TLC (n patients with measurement)	0.75 ± 0.12 (62)

Set of predicted values are quoted in the first column.

*: among these 62 patients, 53 (85%) depicted a restrictive defect defined by VA below the 5^th^ percentile value of TLC minus 150 mL.

## Results

### Cross-sectional assessment

During the six-year period (2007 to 2012), the Clinical Database Warehouse showed that 12 775 LFT (spirometry and plethysmography) were performed and that a diagnosis of NSP was recorded for 841 LFT (6.6%, 95% CI: 6.2 to 7.0%, after excluding LFT with insufficient quality criteria). The selection process is summarized in Figure 1.

Using ICD-10 and CCAM (for bilateral lung transplantation [BLT]) codes, the prevalence of NSP (341 patients) in each lung disease was estimated: asthma, 124/986 (12.6%, 95% CI: 10.5 to 14.6%), COPD/emphysema, 71/828 (8.6%, 95% CI: 6.7 to 10.5%), bronchiectasis, 55/431 (12.8%, 95% CI: 9.6 to 15.9%), sarcoidosis 22/205 (10.7%, 95% CI: 6.5 to 15.0%), interstitial pneumonia 19/475 (4.0%, 95% CI: 2.2 to 5.8%), pulmonary hypertension, 33/369 (8.9%, 95% CI: 6.0 to 11.9%) and BLT for cystic fibrosis, 36/100 (36.0%, 95% CI: 26.5 to 45.5%).

The final selection of 185 Caucasian patients with NSP allowed the description of their characteristics in Tables 1 and 2. The results of the LFT demonstrated that a moderate mean obstructive defect was evidenced associated with mild air trapping. The degree of the obstructive defect was similar whatever the underlying disease whereas the degree of lung air trapping varied among diseases (Table 2). Asthmatics demonstrated the highest BMI values (Table 2), and 31/67 (46%) patients were obese (BMI ≥30 kg.m^−2^).

**Table 2:** **Main characteristics of the patients with NSP according to underlying condition**

Characteristic	Asthma	COPD/emphysema	Bronchiectasis	Sarcoidosis	Interstitial pneumonia	Pulmonary hypertension	Bilateral lung transplantation	***P***value
Mean ± SD (except when stated) or n								
	N = 67	N = 55	N = 14	N = 12	N = 15	N = 7	N = 15	
Sex, F/M	48/19	17/38	12/2	7/5	6/9	5/2	4/11	<0.0001
Age, years	51 ± 15	64 ± 10	60 ± 16	47 ± 15	66 ± 14	47 ± 17	28 ± 9	<0.0001
BMI, kg.m^−2^	30.6 ± 9.3	26.7 ± 6.2	21.1 ± 2.4	27.3 ± 5.3	28.1 ± 5.9	22.4 ± 4.6	17.7 ± 1.6	<0.0001
Smoking history								<0.0001
Never smokers, n	54	0	11	9	10	6	15	
Ex-smokers, n	6	31	2	3	5	1	0	
Current smokers, n	7	24	1	0	0	0	0	
Pack-year, median [IQ]	0 [0 – 0]	45 [36 – 59]	0 [0 – 0]	0 [0 – 4]	0 [0 – 3]	0 [0 – 0]	0 [0 – 0]	<0.0001
MRC score, median [IQ]	2 [1 – 2]	2 [1 – 2]	1 [1 – 2]	2 [2 – 2]	2 [2 – 2]	2 [1 – 5]	1 [1 – 1]	0.0003
FEV_1_,% predicted	64 ± 7	63 ± 8	61 ± 9	67 ± 8	69 ± 8	59 ± 5	66 ± 8	0.118
FVC,% predicted	65 ± 8	65 ± 9	63 ± 9	70 ± 10	68 ± 9	63 ± 5	66 ± 9	0.239
FEV_1_/FVC,% predicted	97 ± 8	96 ± 7	96 ± 7	95 ± 6	99 ± 10	93 ± 5	100 ± 8	0.321
TLC,% predicted	94 ± 10	99 ± 14	96 ± 8	90 ± 7	90 ± 9	96 ± 15	93 ± 6	0.189
FRC,% predicted	102 ± 27	121 ± 26	116 ± 21	97 ± 14	96 ± 16	111 ± 35	127 ± 11	<0.0001
RV,% predicted	132 ± 33	145 ± 33	136 ± 22	119 ± 21	110 ± 25	136 ± 30	169 ± 28	<0.0001

IQ denotes interquartile.

Patients with pulmonary hypertension were classified according to the World Health Organization Groups of pulmonary hypertension [15] as: group 1, n = 3; group 4, n = 2, group 5, n = 2.

### Follow-up study

Among the 841 LFT evidencing a NSP, 360 patients are described in Figure 1 while 30 additional patients were suffering from miscellaneous diseases (see Figure 1 legend). Thus the 451 remaining LFT were related to the patients having a NSP on recurrent tests. Among the 185 patients with indisputable diagnoses, the majority (130/185: 70% 95% CI: 64 to 77%) demonstrated a stable pattern while 16 patients (9%, 95% CI: 5 to 13%) showed a restrictive pattern (TLC < 5^th^ percentile, 12 patients with interstitial pneumonia and 4 with sarcoidosis) and 39 patients (21%, 95% CI: 15 to 27%) showed an obstructive pattern (FEV_1_/VC < 5^th^ percentile, 10 patients with asthma and 29 with COPD/emphysema).

## Discussion

In the presence of a normal TLC, a decrease in VC, and therefore of FEV_1_, is the consequence of an increase in RV. Pathologic conditions associated with intrinsic and extrinsic obstruction of small airways, together with expiratory muscle weakness, will lead to an increased RV. Dynamic expiratory airflow obstruction could also produce an increase in RV and a decrease in FVC. However, measuring a slow VC instead of a forced one would avoid the rise in RV, which was not observed in our study. Consequently, the pattern of NSP may effectively be related to patchy collapse of small airways early in exhalation, and does not seem to be related to a bronchoconstrictor effect of deep inspiration [16] that would be associated with normal slow inspiratory vital capacity.

To our best knowledge, only one study evaluated the main lung diseases that are associated with this pattern [4]. In the retrospective study of Hyatt and colleagues, NSP was observed in 7702/80 929 subjects (9.5%), and a random sample of 100 patients allowed to define the medical conditions associated with this pattern [4]. The authors showed that 52 patients had some degree of airway responsiveness (positive response to bronchodilator or methacholine) including 26 asthmatics, 16 were suffering from chronic lung disease (including 11 with COPD and 3 with bronchiectasis), 7 were obese only and 25 were suffering from various conditions (prevalence ≤5% for each condition). Overall, in their study, obesity (BMI ≥ 30 kg/m^2^) was very prevalent (50 subjects equally distributed between the sexes) and they highlighted the association of NSP with obesity and hyperresponsiveness (31%). Obesity has also been associated with propensity of distal airway closure/hyperresponsiveness [17]. We similarly observed that asthmatic patients in our series were often obese, even if obesity prevalence was lower in our series (26%), which may further explain our lower prevalence of NSP. These authors emphasized that some patients had no evidence of airway disease. Restricted expansion of the thorax or lung may have explained the NSP in most of these subjects since the conditions were suggestive of restriction despite the low normal TLC. Interestingly, some of these conditions have also been associated with increased airway responsiveness [18, 19], which may have favoured the occurrence of this NSP.

Our design had both similarities and differences. Large databases were used in the two studies that used body plethysmography for absolute lung volumes measurements. We deliberately decided not to include all medical conditions associated with NSP (30 patients were excluded) because one objective was to provide confident prevalences of the functional pattern in the various medical conditions. The prevalence of NSP was thus described for seven conditions associated with the pattern, over 360 patients. Then we further selected 185 patients in order to describe the functional abnormalities and to evaluate whether the severity of the pattern varied among the seven conditions. To this end, we selected only Caucasian (to use only one set of predicted values) and non or light smokers in non COPD/emphysema conditions (to exclude overlapping conditions). In agreement with the study of Hyatt and colleagues, a moderate impairment was evidenced (FEV_1_ 68 ± 9% predicted in their study) [4].

A large series of patients has been obtained with indisputable diagnoses and, besides lung diseases already associated with NSP [4], we further show that some conditions as interstitial pneumonia, pulmonary hypertension or bilateral lung transplantation are associated with low to moderate prevalence of the syndrome. Interestingly, in patients with bronchiolitis obliterans syndrome after allogeneic hematopoietic stem cell transplantation [20], Bergeron and colleagues identified two functional phenotypes: a typical obstructive lung defect and an atypical obstructive lung defect with a concomitant decrease in the FEV_1_ and FVC with a normal total lung capacity (31% of the patients, 95% CI: 21 to 42%). Consequently, this latter prevalence is similar to ours, and one may hypothesize that this high prevalence is specifically related to obliterative bronchiolitis [21] rather than to biases related to the presence of lung size mismatching between donor and recipient, and/or inaccurate predicted values for donor lungs.

Before the more recent ATS/ERS Task Force recommendations [1], previous international guidelines did not formally classified this functional phenotype as an obstructive defect, as pointed out by Stanescu [2]. In patients with established airway diseases, as COPD or asthma, a reduction in FEV_1_ would be regarded by most physicians as indicative of airway obstruction whatever the FVC value. On the other hand, when the diagnosis has not been established or in presence of a patient with pulmonary hypertension, a “restrictive” pattern observed after the sole spirometry may lead to misdiagnosis, accordingly with the low predictive value of spirometry for lung restriction [22]. Furthermore, measurement of absolute volumes by dilution technique may lead to misdiagnosis (restrictive pattern), as suggested by the reduction of alveolar volume as compared to TLC, which would lead to restrictive defect diagnosis in 85% of the patients (see Table 1).

Some authors prefer to label this pattern as non specific due to the normalcy of the FEV_1_/VC ratio and of the TLC [4, 5]. Nevertheless, it has to be emphasized that small airway (bronchiolar) involvement is a characteristic or can occur in all these diseases. For instance in pulmonary hypertension, a mild obstructive pattern is well-described that can be associated with exercise-induced dynamic hyperinflation [23]. Small airway disease is also well demonstrated in interstitial pneumonias [24], suggesting a mixed defect when an additional restrictive defect occurs as observed in our study. The mild to moderate lung air trapping that was evidenced in our study together with an increase in airway resistance further suggest distal airway obstruction. Along this line we showed that both airway resistance and specific airway resistance can augment in the presence of peripheral airway obstruction [25, 26].

Finally, the observed prevalence of this syndrome in selected conditions may seem relatively high, but we previously demonstrated that isolated hyperinflation is not infrequent in asthmatic children (7-11%) [3]. Logically, due to this prevalence in common respiratory diseases, the overall prevalence in our LFT unit (6.6%) is in agreement with the results of Hyatt and colleagues (9.5%) and with those of Aaron and colleagues (15%, 95% CI: 13 to 17% patients with low FVC and normal absolute lung volumes) [22]. Unfortunately, the diagnoses associated with this specific functional pattern were not described in this latter study [22].

Our study has inherent limitations due to its design. Data extraction was based on the actual recording of NSP by the physician in charge of the LFT report made in the routine practice; consequently, the observed prevalences could have been underestimated. These prevalences are only indicative because some patients had several LFT that may have facilitated the detection of NSP, which can be a transient functional phenotype [5]. Reversibility of airway limitation was not systematically assessed because patients are tested while taking their usual respiratory treatment. Finally, our study was not designed to evaluate all lung diseases associated with NSP, since only concordant and indisputable diagnoses were retrieved for functional description. Whether NSP is associated with specific clinical phenotypes warrants further studies.

## Conclusion

In conclusion, NSP can be observed in asthma, COPD/emphysema, bronchiectasis, sarcoidosis, interstitial pneumonias, pulmonary hypertension and after bilateral lung transplantation. Thus, the measurement of static lung volumes by body plethysmography can be helpful in presence of FEV_1_ and FVC reduction, depending on previous measurements, lung imaging, and clinical judgment.
